# Embodied emotions in ancient Neo-Assyrian texts revealed by bodily mapping of emotional semantics

**DOI:** 10.1016/j.isci.2024.111365

**Published:** 2024-12-04

**Authors:** Juha M. Lahnakoski, Ellie Bennett, Lauri Nummenmaa, Ulrike Steinert, Mikko Sams, Saana Svärd

**Affiliations:** 1 Institute of Neuroscience and Medicine, Brain & Behaviour (INM-7), Research Center Jülich, Jülich, Germany; 2 Institute of Systems Neuroscience, Medical Faculty, Heinrich Heine University Düsseldorf, Düsseldorf, Germany; 3 LVR-Klinikum Düsseldorf, Heinrich-Heine University Düsseldorf, Düsseldorf, Germany; 4 Centre of Excellence in Ancient Near Eastern Empires (ANEE), University of Helsinki, Helsinki, Finland; 5 Turku PET Centre, University of Turku and Turku University Hospital, Turku, Finland; 6 Department of Psychology, University of Turku, Turku, Finland; 7 Johannes Gutenberg-University Mainz, Mainz, Germany; 8 Department of Neuroscience and Biomedical Engineering, Aalto University, Espoo, Finland; 9 MAGICS–Aalto, Aalto University, Espoo, Finland

**Keywords:** Neuroscience, Linguistics, History

## Abstract

Emotions are associated with subjective emotion-specific bodily sensations. Here, we utilized this relationship and computational linguistic methods to map a representation of emotions in ancient texts. We analyzed Neo-Assyrian texts from 934–612 BCE to discern consistent relationships between linguistic expressions related to both emotions and bodily sensations. We then computed statistical regularities between emotion terms and words referring to body parts and back-projected the resulting emotion-body part relationships on a body template, yielding bodily sensation maps for the emotions. We found consistent embodied patterns for 18 distinct emotions. Hierarchical clustering revealed four main clusters of bodily emotion categories, two clusters of mainly *positive* emotions, one large cluster of mainly *negative* emotions, and one of *empathy* and *schadenfreude*. These results reveal the historical use of embodied language pertaining to human emotions. Our data-driven tool could enable future comparisons of textual embodiment patterns across different languages and cultures across time.

## Highlights

• We summarize co-occurrence of body and emotion words in Neo-Assyrian texts from 934–612 BCE
• We create body maps to visualize how embodied emotions were described in ancient Mesopotamia
• The body maps demonstrate clusters of emotions with similar bodily representations
• These maps may enable comparison of embodied emotion between different eras and cultures

## Graphical abstract

**Figure:**
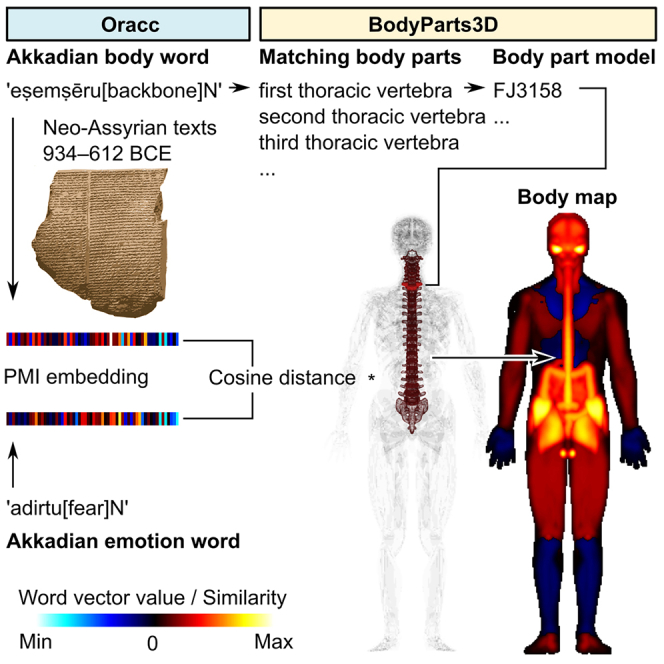

## Introduction

Emotions are associated with autonomic-activation-triggering subjective sensations throughout the body. Physiological changes, such as elevated heart rate, sweating, and contractions of facial muscles can be observed and measured to quantify bodily responses to emotions.^1^ These physiological changes contribute to the subjective emotional state and form the basis of our subjective experience of emotion.^2^

Subjective bodily sensations during emotions are consistent across cultures,^3^ suggesting a shared biological basis of emotions. A bulk of studies using topographical self-reports of embodied feelings has revealed culturally consistent topographies in emotions evoked by mental imagery, videos, texts, and music.^1^^,^^3^^,^^4^^,^^5^ In these studies, the subjects were presented with silhouettes of human bodies and indicated by “painting” with a mouse the regions where they felt changes during the evoked emotion. Unfortunately, self-report and laboratory studies can only assess the inter-individual similarity of subjective feelings in the current world and not, e.g., for long-dead individuals from historical cultures. However, establishing statistical regularities between emotional expressions (e.g., “I am happy”) and references to bodily regions or organs (e.g., “my heart felt light”) in historical textual sources would provide a means for establishing the embodiment of emotions in ancient texts. Moreover, such text-to-body mapping would allow comparison of bodily emotional representations in historical sources vs*.* modern human subjects.

To evaluate how individuals in distant cultures—in time, geography, and customs—may have experienced emotions, it is possible to analyze emotion expressions in written documents, which have been dutifully compiled and analyzed in many languages.^6^ Semantic analysis of text corpora from the premodern era can give us a glimpse into the emotional lives of civilizations that have long ago ceased to be.^7^^,^^8^^,^^9^ Comparison of emotion expressions across languages is however difficult. Here, we propose and demonstrate an approach for computational language studies by quantifying how textual descriptors of emotions map into the body, based on the proximity of emotion terms or expressions and words referring to specific bodily parts in the texts. This allows for a comparison with recent research, contributing to neuroscientific, cognitive, and historical discussions about embodied emotions.

We focus on a corpus of texts from the Neo-Assyrian period written in Akkadian, a Semitic language. The Neo-Assyrian period is characterized by a rapid expansion of territory controlled by Assyrians in the north of what is now modern-day Iraq from 934–612 BCE. At its height, the Assyrians controlled territory from the Zagros mountains to Egypt and Eastern Turkey.^10^ This huge empire left behind thousands of cuneiform texts in the Akkadian language in various genres that have been published in text editions by scholars. The quantity and diversity of preserved texts from this period makes this an excellent candidate for exploring the methodology set out in this article.

The study of emotions in texts written in Akkadian is a recent development and has focused on individual lexemes, domains of emotions, or specific text genres.^7^^,^^11^^,^^12^^,^^13^^,^^14^^,^^15^ The specific topic of the embodiment of emotions in Akkadian has only recently been discussed.^16^^,^^17^ These studies have revealed the frequent references to body-related processes of emotional experiences, including how Akkadian considers internal organs as seats (“containers”) of emotions or embodied feelings. For example, the terms *libbu* (a word that can vaguely mean “inside [of the body, torso]” but can also more specifically refer to the belly, the heart, or the womb) and *kabattu* (“liver; innards”) are frequent in descriptions of embodied feelings and emotion expressions.^11^^,^^12^^,^^13^^,^^17^^,^^18^ Steinert^16^ also observed that some of the patterns regarding where Akkadian emotions (such as *Joy*, *Love*, *Pride*, *Disgust*, *Contempt*, *Sadness*, and *Depression*) were located in the body correlated with the bodily sensation maps elucidated through neuroscientific emotion research.^1^ Even though the locations of embodied emotions were similar in these modern neuroscientific studies, there were culturally specific conceptual metaphors in Akkadian texts used to express these embodied emotions.^17^ As the study of embodied emotions is still in its infancy for the Neo-Assyrian period, our results will enhance the growing field in an important way.

Most of the work that has been done on history of emotions and the body within the field of ancient Near Eastern studies has relied on qualitative and philological methods. However, the digital editions of Akkadian texts available in open digital projects such as the Open Richly Annotated Cuneiform Corpus (Oracc; http://www.oracc.org) have led to developments of Natural Language Processing (NLP) and corpus-linguistics-based methods for Akkadian data. Many are based on the methods developed by corpus linguists working with modern language corpora, but methods such as word co-occurrences and vector space analysis that have been tailored to the unique challenges of Akkadian datasets (such as a lack of punctuation and a tendency to be highly repetitive) have recently been developed.^8^^,^^19^ These tools can now be used to explore complex topics, such as cultural associations of specific words or groups of words as well as emotion expressions in languages in the ancient Near East.^7^^,^^8^^,^^14^^,^^20^^,^^21^

**Figure 1:**
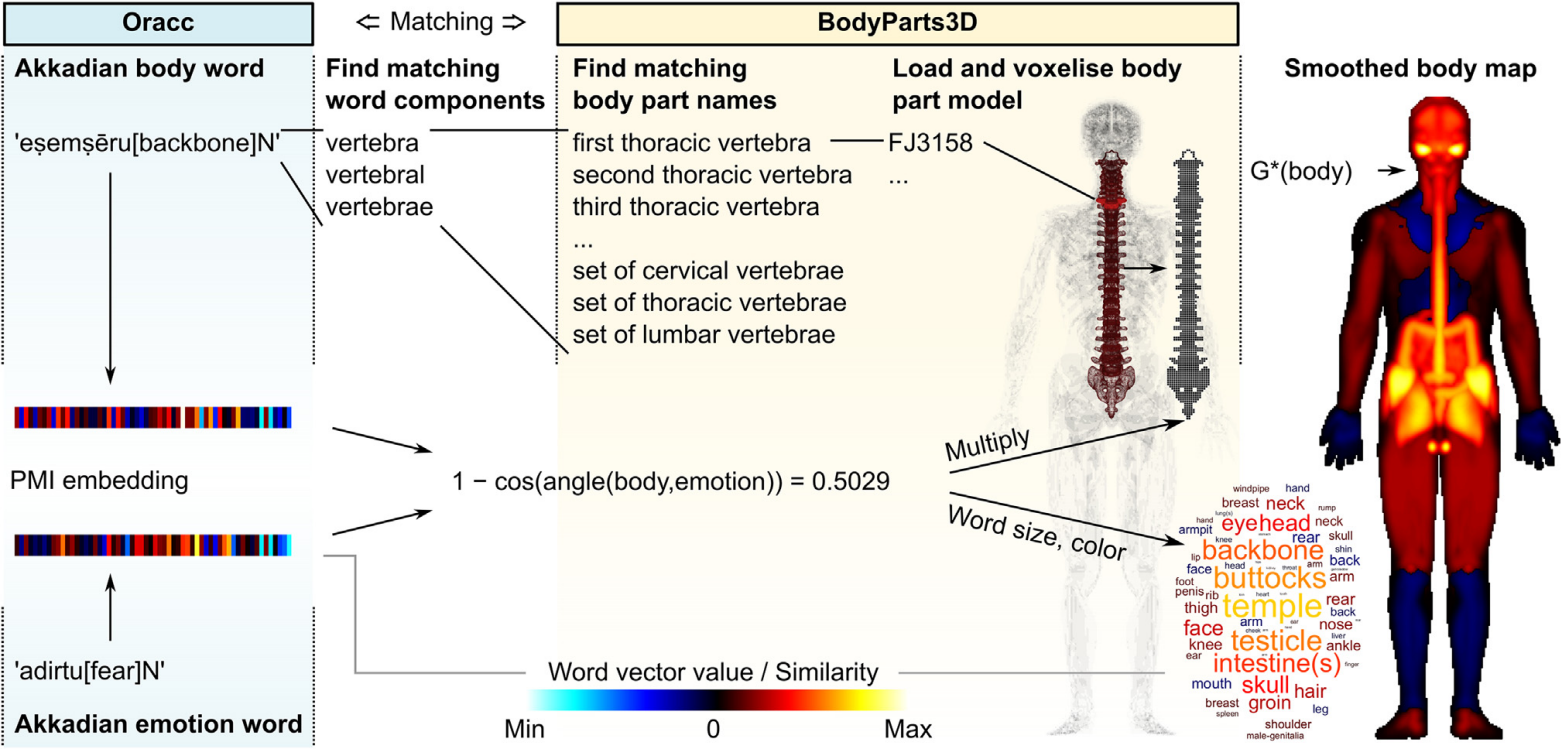
Emotion visualization pipeline To match a body word from the Oracc corpus (pale blue, left) to its anatomical model, unique word components were searched from the BodyParts3D model hierarchies (mid left). The word components was expanded to the list of models that included the component words in their name and all models that belonged to the matching compound body part lower in the hierarchy (pale yellow, mid right). The models comprising the body part were then loaded and a combined voxelized logical body part model was created with ones indicating the volume contained within the model and zeros outside. To produce the emotion maps, each model was assigned the similarity value between the emotion and body word pair by multiplying the logical body map by the cosine similarity of the body part and emotion word in the PMI embedding word vector space, and the body parts were combined into a complete body map based on the local (positive or negative) extreme values of overlapping anatomical regions that was smoothed with a Gaussian kernel for visualization.

In the present proof-of-concept study, we suggest a method for retrieving quantitative associations between references to specific body parts and distinct emotions in Akkadian texts. We then show how these associations can be mapped as sensation topographies in the whole body, similar to what has been done in modern large-scale studies on bodily basis of emotions with living participants.^1^^,^^3^^,^^4^^,^^22^ We do this by first identifying all the words in the Neo-Assyrian corpus taken from Oracc that could be used to describe either emotions or parts of the body. We then summarize the co-occurrences of the words in the corpus using point-wise mutual information (PMI) embeddings to derive a low dimensional representation of the co-occurrence patterns. To visualize the co-occurrence of body and emotion words, we create three-dimensional maps of the body parts mentioned in the corpus using the BodyParts3D open access library of models of the human body parts by matching the Akkadian body words with the body part models in the library. We then use the cosine similarity between word embeddings of body-emotion word pairs to modulate the color intensity of the body part models to create easily interpretable maps representing the body-emotion associations in the literature. The process is visualized in Figure 1 and explained in detail in STAR Methods section. Finally, we evaluate the similarity between and within previously identified emotion categories to derive a hierarchy of embodied emotions in the Akkadian language. The emotion categories and number of individual emotions in each category before and after word exclusion are shown in Table 1. For details on word exclusion criteria, see STAR Methods. We believe these methods and results may be used for comparative studies with written corpora of different time periods and cultures.

**Table 1:** Emotional categories as identified by the AHEC team

Primary emotion category	Number of words	Number of words after exclusion
1. a) Sadness, grief, depression, melancholy	56	47
1. b) Distress (sadness and worry)	21	16
1. c) Suffering	16	14
2. Anger	62	53
3.a) Happiness, joy	44	35
3. b) Schadenfreude	3	2
3. c) Pleasure (as part of Joy)	2	2
4. Fear, anxiety, panic, nervousness, awe, respect	47	37
5. Despise, hate, contempt	18	17
6. a) Love, affection, admiration	13	9
6. b) Desire (as a part of love)	6	6
7. Disgust	6	5
8. Sympathy, compassion, pity	10	7
9. Envy, jealousy	1	1
10. Pride	16	15
11. Surprise	2	2
12. Shame, embarrassment	2	1
13. Sexual arousal	6	6

The columns list the names of the emotional categories and their hierarchical structure (e.g., 1a, 1b, …) as well as the number of words (individual lexemes, not the total count of word occurrences in our corpus) assigned to each category and the number of words included in the results of the current study. The process for excluding words is discussed in more detail in the section “word embeddings” in STAR Methods. The underlined section of the emotional category indicates how it is labeled and discussed in the results and discussion sections.

## Results

### Emotional categories on the body

**Figure 2:**
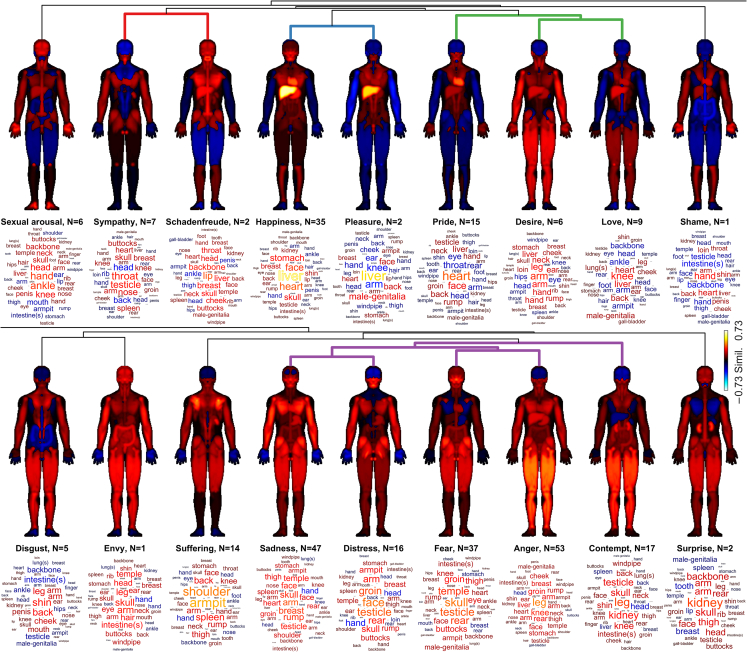
Mean co-occurrence patterns of emotions included in the 18 principal emotional categories across the body Each emotional category is represented by a body map (top) and a word cloud (bottom). The number of emotion words in each category is indicated by “N = ”. Red colors indicate high similarity of word embeddings between body and emotion words, blue color indicate anti-correlated word embeddings, and black indicates a lack of statistical association. In the word clouds, the strength of association for specific body parts is indicated by the font size in addition to the text color. Clustering of emotion categories is indicated on top of the body maps with the branches of the linkage tree from Figures 3 and 4.

**Figure 3:**
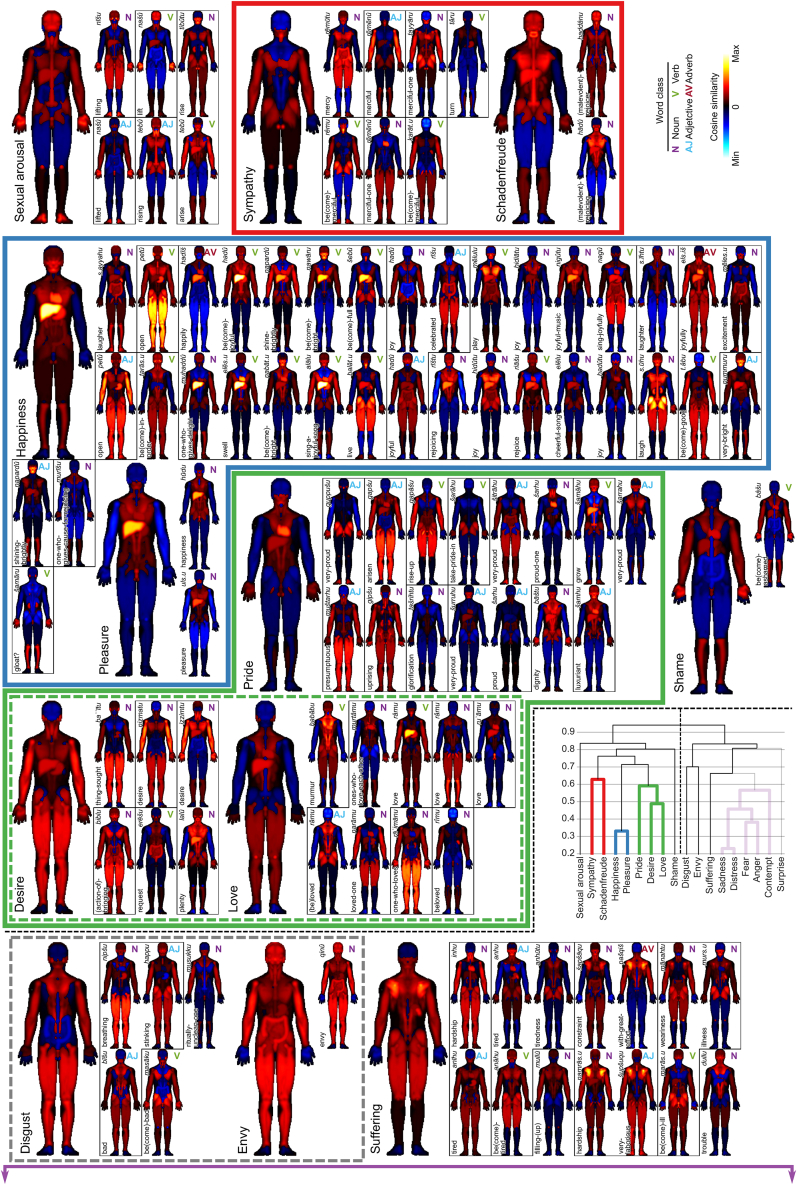
Bodily maps of individual emotions within primary emotional categories of mainly positive emotions Emotional categories are grouped according to weighted average linkage clustering (linkage tree on the right; y-axis corresponds to the linkage values) based on the cosine distance of the mean emotion vectors of the primary emotional categories. The excluded branches are shown in transparent shades in the tree. The individual emotions are ordered based on inter-emotion distance on similar clustering trees within emotion clusters. Word classes are indicated by the colored abbreviations at the top right of each emotion map. Clusters with mainly positive emotions are shown in this figure. The clusters including mainly negative emotions are shown in Figure 4. Subclusters (branches) are surrounded by colored (gray) dashed outlines. Black dashed line in (A) indicates the split point between the two main branches of the linkage tree. Borders of the sub-clusters within an emotion category are indicated by black outlines, whereas emotions falling outside of clusters are shown without outline.

**Figure 4:**
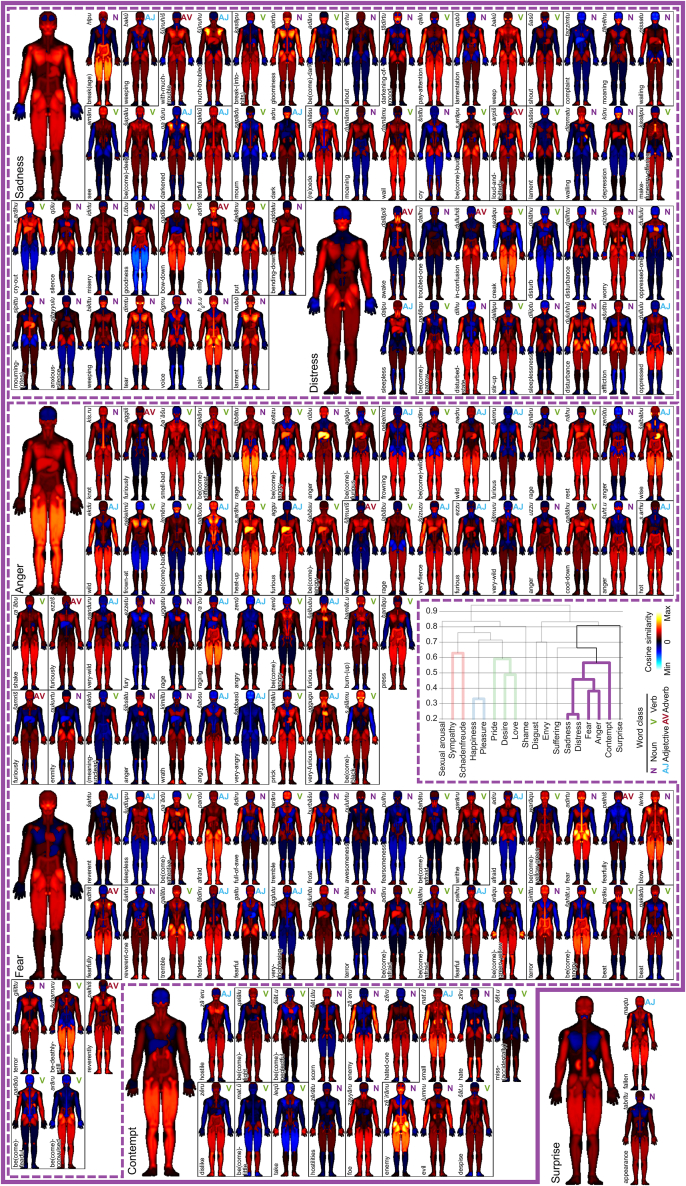
Bodily maps of individual emotions within primary emotional categories of mainly negative emotions The organization follows the description of Figure 3.

The body maps for the 18 emotion categories are shown in Figure 2 with the names of the emotion category and the number of the emotion words included in the category reported below the body maps. The emotions are ordered according to the similarity-based clustering of the body maps between the emotions (indicated by the linkage tree on top of the body maps; see also Figures 3 and 4). To illustrate the specific body parts showing highest similarity for the emotional categories, the English names of the body parts are additionally represented as word clouds under the body maps, where the font size and color correspond to the similarity value. The five body parts with highest similarity for each emotional category are listed in Table 2.

**Table 2:** Top five most important body words for the primary emotional categories

Sexual arousal	Sympathy	Schadenfreude	Happiness	Pleasure	Pride
eṣemṣēru[ankle]N	napištu[throat]N	šaptu[lip]N	kabattu[liver]N	kabattu[liver]N	nupāru[heart]N
0.33	0.3	0.36	0.67	0.51	0.37
qātu[hand]N	išku[testicle]N	eṣemṣēru[backbone]N	nupāru[heart]N	lipištu[male-genitalia]N	zīmu[face]N
0.33	0.3	0.3	0.37	0.25	0.25
qaqqadu[head]N	appu[nose]N	tulû[breast]N	zīmu[face]N	ṣēru[back]N	ṣēru[back]N
0.27	0.23	0.29	0.34	0.22	0.18
birku[knee]N	gulgullu[skull]N	kabattu[liver]N	karšu[stomach]N	zīmu[face]N	kabattu[liver]N
0.26	0.22	0.27	0.29	0.2	0.18
tikku[neck]N	idu[arm]N	gulgullu[skull]N	gulgullu[skull]N	nupāru[heart]N	šuburru[rump]N
0.22	0.21	0.27	0.26	0.18	0.17

**Desire**	**Love**	**Shame**	**Disgust**	**Envy**	**Suffering**

idu[arm]N	burku[knee]N	qātu[hand]N	kimṣu[shin]N	ahu[arm]N	šahātu[armpit]N
0.32	0.29	0.29	0.29	0.31	0.46
purīdu[leg]N	kabattu[liver]N	nupāru[heart]N	kutallu[back]N	gulgullu[skull]N	būdu[shoulder]N
0.26	0.24	0.23	0.26	0.3	0.41
durāʾu[arm]N	nupāru[heart]N	kimṣu[shin]N	purīdu[leg]N	purīdu[leg]N	ṭulīmu[spleen]N
0.26	0.2	0.2	0.25	0.28	0.29
kappu[hand]N	kutallu[back]N	zāqu[arm]N	lētu[cheek]N	šārtu[hair]N	kutallu[back]N
0.23	0.19	0.19	0.22	0.27	0.25
kabattu[liver]N	lipištu[male-genitalia]N	kutallu[back]N	ahu[arm]N	qaqqadu[head]N	šuburru[rump]N
0.23	0.18	0.18	0.22	0.27	0.22

**Sadness**	**Distress**	**Fear**	**Anger**	**Contempt**	**Surprise**

ahu[arm]N	pānu[face]N	gulgullu[skull]N	purīdu[leg]N	purīdu[leg]N	kalītu[kidney]N
0.36	0.39	0.39	0.41	0.34	0.34
tulû[breast]N	išku[testicle]N	išku[testicle]N	birku[knee]N	kalītu[kidney]N	gulgullu[skull]N
0.34	0.37	0.36	0.31	0.29	0.24
šuburru[rump]N	šapūlu[groin]N	kutallu[rear]N	šapūlu[groin]N	išku[testicle]N	sīqu[thigh]N
0.33	0.35	0.33	0.26	0.25	0.24
gulgullu[skull]N	kutallu[rear]N	usukku[temple]N	kutallu[rear]N	gulgullu[skull]N	durāʾu[arm]N
0.32	0.31	0.32	0.25	0.25	0.23
išku[testicle]N	ahu[arm]N	šapūlu[groin]N	idu[arm]N	burku[knee]N	ahu[arm]N
0.31	0.29	0.28	0.25	0.24	0.23

The values correspond to the cosine similarity of the mean vector of the emotion category and the body part vector.

To evaluate the similarity of the resulting body maps, we performed a two-stage hierarchical weighted average linkage clustering of the emotion categories and the individual emotions (Figures 3 and 4). The top-level clustering reflects how similar the body maps of each emotion category (averaged across all sub-emotions belonging to the primary emotional category) are to the others. The linkage value between two clusters reflects the weighted average cosine distance of all pairs of primary emotional categories between the clusters and the levels where the tree branches indicate the weighted average pairwise cosine distance at which two clusters are joined. Lower linkage values indicate higher similarity between clusters, and the cluster threshold is set to the MATLAB default setting of 70% of the maximum value observed in the current data.

The clustering solution revealed four consistent clusters. The first included the emotions of *Sympathy* and *Schadenfreude*, the next two clusters consisted mainly of positive emotions (Figure 3), one comprising the emotions *Happiness* and *Pleasure* and the other consisting of the emotions *Pride*, *Desire*, and *Love*. The final, large cluster (Figure 4) consisted of the emotions *Sadness*, *Distress*, *Fear*, *Anger*, and *Contempt*. The colored outlines around emotion categories in Figures 3 and 4 indicate the clustering solution. *Sexual arousal* and *Shame* fell into the branch containing the two mainly positive emotions but were not included in any cluster. *Disgust*, *Envy*, *Suffering*, and *Surprise*, in turn, fell into the negative emotions branch of the linkage tree but were not included in any of the main clusters. The word classes of the Akkadian emotion words in Figures 3 and 4 (verb, noun, adjective, or adverb) are indicated by the colored abbreviations at the top right corner of each small body map.

The individual emotions within each primary emotional category in Figures 3 and 4 are ordered based on their pairwise similarities in a secondary average linkage clustering, so that individual emotions belonging to the same sub-cluster are shown next to each other. Borders of the sub-clusters are indicated by black outlines, whereas emotions falling outside of clusters are shown without outline.

## Discussion

Our main finding was that specific body parts (such as the internal organs) are consistently highlighted in the body maps of the 18 primary emotion categories in the Akkadian language. These mappings also contain meaningful hierarchical structure, in that the bodily maps of emotion-body part correspondences cluster into four broad emotion categories: two primarily positive clusters, one large negative cluster, and one related to empathy toward others, or lack thereof. Importantly, this “corporal” representation of emotions in the Akkadian language provides an intuitive understanding of how emotions are embodied in the texts and yields a more directly interpretable way to compare between emotions than the raw word vectors derived from text corpora.

### Bodily maps of emotional categories in Neo-Assyrian textual corpus

The 18 primary emotional categories included in the current analyses were associated with distinct spatial patterns on the body (Figure 2). The primary emotions also clustered into four main clusters and two main branches, broadly following a positive/negative division of emotions as usually found in emotion research of contemporary societies.

Cluster 1 included the emotions *Sympathy* and *Schadenfreude*. These emotions exhibited common positive cosine similarity values for the torso, particularly in the chest area, as well as in the throat and lower parts of the face. By contrast, the limbs and upper part of the head showed different patterns between these two emotions.

Cluster 2 included the emotions of *Pleasure* and *Happiness*. In the two averaged body maps, the liver was the body part with the highest cosine similarity value. However, when investigating the individual body maps, this association was the most consistent and strong across the words for *Happiness*.

Cluster 3 includes the emotions *Pride*, *Desire*, and *Love*. Across all these body maps, there was a positive cosine similarity score between two words for key internal organs (the liver and the heart) and the words for all three of these emotions. In addition, all three general body maps have a negative cosine similarity value for the head and hips. However, the sub-group of *Desire* and *Love* had an additional similarity of positive cosine similarity scores for the legs as well as internal organs.

Clusters 1, 2, and 3 were part of the same larger branch that included *Sexual arousal* and *Shame*, which also exhibited positive similarities with the heart and liver.

Cluster 4 is the largest cluster and includes the emotions *Sadness*, *Fear*, *Anger*, *Contempt*, and *Distress*. *Sadness* and *Distress* were in the same sub-group of the cluster, likely because only the hands and upper head for both emotions had negative cosine values—the rest were positive. The averaged body maps for *Anger* and *Fear* shared a common positive cosine score for the thighs and upper legs and as such were part of another sub-group of Cluster 4. The final emotion in this cluster, *Contempt*, had an interesting negative cosine value for the liver.

In addition, Cluster 4 was in a common branch of the individual emotions of *Disgust*, *Envy*, *Suffering*, and *Surprise*. Although each of the body maps for these emotions demonstrate differences from each other, all four continue the theme of the limbs and internal organs as having positive cosine similarities. The clustering of emotion categories was broadly in line with the hierarchy of AHEC categories, with some notable differences. For instance, suffering (1c) did not collocate in the same lowest-level cluster as its companion emotions Sadness (1a) and Distress (1b) but was rather a separate leaf in the “negative branch” of the clustering tree. Similarly, schadenfreude (3b) clustered with sympathy (8) rather than the other emotions in the broader emotional field, namely happiness (3a) and pleasure (3c), which stayed together in the data-driven clustering. Moreover, the hierarchical cluster analysis of Akkadian emotions highlights some remarkable similarities to cluster analyses of emotion (terms) in modern emotion research based on empirical data. For example, analyses of 24 selected emotions applying the so-called GRID instrument identified that emotions are organized in two higher-order clusters (a positive and a negative one) and four major emotion clusters around the focal emotions of *Joy*, *Fear*, *Sadness*, and *Anger.* The emotions of *Surprise* and *Compassion* formed a separate cluster, similar to the emotions of *Sympathy* and *Schadenfreude* in the present study (which may be due to the ambivalent valence of these emotions in the respective cultures). This consistency in focal emotion clusters suggests that there are some consistent patterns of (dis)similarity between emotion categories expressed in different languages that are also detected or picked up when one focuses on the specific aspect of emotion embodiment.^23^

The consistent emphasis of internal organs and limbs can also be seen in Table 2, where 12 of the top 2 results for the emotional categories are words relating to limbs and 8 relate to internal organs. This finding broadly aligns with the results of previous Assyriological research on (embodied) emotions, particularly in discussions of *Happiness*,^11^^,^^12^^,^^13^ *Fear*,^7^ and *Anger*.^24^ For example, the liver has been discussed by scholars as the seat of anger and happiness in Neo-Assyrian texts.^11^^,^^12^^,^^13^^,^^16^^,^^24^ However, several of the categories identified by the AHEC team have not been studied in as much depth, such as *Surprise.* Some words from other categories in the current material are also used in contexts of negative surprise (related to shock, e.g., *šuḫarruru,* here *“be-deathly-still”* but also translated as *“to become dazed, still, numb with fear”*). The results from the body maps for emotions such as these are therefore less clear and will be the focus of future research.

Compared with prior studies on self-reported bodily sensations,^1^^,^^3^ the body-emotion associations in the Neo-Assyrian texts are partly different than those explicitly reported by modern volunteers. There may be several factors affecting this, such as differences in the way emotions were culturally discussed in ancient Mesopotamia compared to modern times, different understandings of anatomy and physiology, or a more general difference in how body and emotion words co-occur in written texts as compared to what people themselves report when reflecting on how they experience emotions in their body. Thus, it should be noted that body part terms in Akkadian texts are used in a variety of expressions highlighting different aspects or components of affective experiences (not only subjective feelings but also physical symptoms or facial/vocal expressions of emotions). Constructions with body part terms may be literal or figurative expressions combined with or without an emotion term (e.g., *muruṣ libbi* “sorrow, literally illness of the heart,” phrases such as “my legs tremble [in fear],” etc.). Moreover, the style of writing may affect how emotions are discussed in general (e.g., personal letters vs*.* official documents), as discussed below in relation to the specific case of “love.” Accordingly, there are Akkadian texts that have not yet been included in the Oracc corpus, which may contain associations that are not reported here.

The emotion categories reflected in Akkadian emotion vocabulary can also be very context-specific in the texts. For example, as demonstrated in recent studies, for modern subjects, love is not a single monolithic entity. Instead, different types of love differentially activate the brain^25^ and people identify different types of love in different parts of their body.^22^ “Passionate love,” “true love,” and “love for life” were mapped in the body as intense sensations covering the whole body. Prototypical “romantic love” and “sexual love” were felt in the torso and in the head of an individual. In the body maps created by Nummenmaa et al. (see Figures 2^1^ and 3,^4^), modern live subjects indicated that “love” (without finer differences indicated) was an intense positive feeling covering roughly torso and head of an individual. When compared with the bodily map of Neo-Assyrian “love” presented in this article (see Figures 3 and 4), the results are somewhat similar. One can see an association between “love” and the torso as well as legs. The relevant body parts (see Table 2) are “burku[knee]N,” “kabattu[liver]N,” “nupāru[heart],” “kutallu[back]N,” and “lipištu[male-genitalia]N.” The association between “love” and “kabattu[liver]N” and “nupāru[heart]N” and “lipištu[male-genitalia]N” seems to match the reported sensations of modern individuals, but the appearance of “burku[knee]N” in the top five words with the closest association to “love” is interesting. These details should be studied in more detail in the future. It could be that the genre of texts would be relevant here, as Akkadian genres tend to use specific vocabulary, and this therefore could mean some words related to emotions may not be as frequent in certain genres. This has already been suggested by a quantitative study of Akkadian concept of “love.”^9^

### Variability of embodiment of emotions within emotion categories

Although the maps for the primary emotional categories and clusterings are clear, there is also considerable variability between bodily maps for individual Akkadian words belonging to the same primary emotion category. For example, the Akkadian words under the emotional field of *Pride* show considerable variability between the body words (Figure 3). This suggests that a deeper analysis focusing on one of these emotions and investigating the words within that field could reveal a more nuanced understanding of how emotions were embodied in Akkadian (and more specifically Neo-Assyrian) texts. Variability within an emotion category could be related to a more complex usage of emotion words than indicated by the principal emotional categories used in the current study, which has been demonstrated by the complex usage of “fear” words in a recent study using similar methods.^7^ Additionally, usage of emotion words could vary with other factors such as the genre of writing or the date when the text was written, among others. This type of visual exploration based on quantitative analysis may enable future research directions in textual corpora, not limited to Akkadian. Moreover, data-driven clustering of the whole emotion space could highlight similarities in the use of emotion words that are not obvious in the manual categorization of emotions. However, more detailed exploration of the full emotion space and comparative work between languages is outside the scope of the current paper focusing on developing the method to enable such visual explorations. Thus, these research directions should be explored further in future work.

### Conclusion

Here, we provide a full-body mapping of emotions and their bodily basis in the Akkadian language. This visualization method enables an intuitive understanding of embodied emotions in historical text corpora and provides a quantitative means for mapping emotions in natural language corpora. The results align with the findings of traditional Assyriological research of embodied emotions but also suggest interesting possibilities of further research. This method can be applied to corpora from different time periods and languages, enabling comparisons of how embodied emotions are discussed across various cultural contexts. This approach aids in both textual and psychological evaluation of embodied emotions throughout history.

### Limitations of the study

In future studies using this method, the size of the textual data must be accounted for. The Neo-Assyrian corpus used for this study is just over one million words, which even though is a small linguistic corpus for modern languages, is comparable in size to other historical text corpora. For example, the Helsinki Corpus of English Texts (https://varieng.helsinki.fi/CoRD/corpora/HelsinkiCorpus/index.html) includes texts from 730–1710 CE and is approximately 1,500,000 words. Thus, the Neo-Assyrian corpus is comparable to other historical corpora in size, enabling quantitative comparisons between corpora in the future.

Several methods have been developed for semantic analysis based on co-occurrence patterns of words in large corpora of texts.^26^^,^^27^ These methods typically produce a lower-dimensional representation (i.e., the process of Truncated Singular Value Decomposition as described in the section “word embeddings”) of the words based on their co-occurrence with other words. However, these representations differ depending on the selected method, and comparison of results produced using different methods may highlight differences in methodology rather than the source material. Moreover, the dimensionality of word representations can be arbitrarily selected even using the same method, which can also affect the resulting maps of emotions on the body (for example, selecting 100 or 200 dimensions instead of 60). Here, we employed the PMI embeddings that have been developed with a specific focus on the Akkadian corpus used in the current study.^8^ Future comparative studies based on our Neo-Assyrian results will show how well this method generalizes to other corpora.

The distance/similarity measures used to summarize the similarity of the representations also affect the results. Here, we used the cosine similarity, which has previously been used with PMI embeddings.^8^ However, with other semantic spaces, some dimensions may dominate the cosine similarity potentially making rank-based similarity measures more robust. Moreover, some semantic spaces may contain a strong shared component between all or most of the words. This may hide distinctions between emotions, making all emotions look relatively similar. Controlling for such shared vector component with methods such as partial correlations may enable stronger distinctions between emotions but, importantly, it may also introduce artificial negative correlations that complicate interpretations as was also demonstrated mathematically in a neuroimaging context.^28^

A further consideration is the limitation of mapping Akkadian words onto the available anatomical models. Although the BodyMaps3D library is one of the most extensive of the available libraries, it only includes male anatomy. Therefore, we could not map the body-emotion associations for Akkadian terms for female genitalia and anatomy onto the library models. This is a non-trivial limitation, as female genitalia and reproductive organs like the uterus were used to express emotions in an ungendered manner in Akkadian.^15^ For example, *rēmu* (“womb”) was used most often in Neo-Assyrian texts to describe the mercy or compassion of the male Assyrian king, such as in one letter where the author described how “Compassion (*rēmu*) took hold of the king” (https://oracc.museum.upenn.edu/saao/saa16/P334623.7.8#P334623.2). In addition, we could not map important words that have more general or amorphous definitions to a single body part or anatomical system. The best example of this is the Akkadian word *libbu*. Despite its recurring use to express emotions in Akkadian texts, *libbu* does not refer to a single organ or body part. Instead, it refers to a general seat of sensations and emotions “inside the torso.”^16^ Although it may have changed the bodily maps around the torso, its general definition meant it proved too ambiguous to map to a limited set of body part. Moreover, another prior study suggested that PMI embeddings highlight the more general usage of the word *libbu* in Akkadian for “inside” locations such as fields or towns, which could have erroneously contributed to the body maps here.^8^ For these reasons, we decided to exclude it in the current study. An interesting future avenue for this research would be to manually create models for the missing anatomy (e.g., *libbu*, vagina, and uterus), and investigate whether they change our present results. Moreover, comparing the way extended bodily systems or bodily humors are discussed in emotional contexts compared to individual bodily organs or regions is an interesting question for further research. Here, we excluded extended bodily systems, such as veins, and general terms that overlapped with more specific ones (e.g., limbs vs. arms). The extended associations over multiple bodily regions in the current results suggest that emotions are indeed not localized to singular regions in the body but span larger systems. Thus, evaluating the excluded words and other broad bodily systems in multilingual material in the future could expand the current findings considerably. Moreover, in the future, direct comparison of the current results to ones derived from modern texts in different languages from different cultures, ideally together with self-reports from the same cultures, would clarify which differences between results are due to different methods and which ones reflect consistent cultural differences.

Finally, the patterns gleaned from PMI embeddings do not reflect the context of the relationship that exists between the body part and the emotion. For instance, statements like “my legs felt weak” and “my legs felt strong” may be associated with different emotions but would be represented by similar amounts of mutual information. Thus, the current body maps are not directly comparable to previous work assessing “activation” and “deactivation,” as both activation and deactivation would conceivably lead to similar co-occurrence patterns in different contexts. Future work could seek to find solutions to disentangling such triangular relationships between emotions, body parts, and associated adjectives and verbs that could reflect differences in the activation dimension of the associations. One option may be to focus directly on the collocates of emotion and body words within Akkadian texts, where the focus is on which other words co-occur close to emotion or body words. Such an approach has already begun to be explored with studies focusing on cultural elements like seeing, age and masculinities, and love have already been carried out in Assyriology.^9^^,^^14^^,^^20^ This would enable direct exploration of which other words occur with each emotion-body word pair. Additionally, the recent advances in generative language models may extend corpus-based methods in the future.^21^^,^^29^^,^^30^^,^^31^^,^^32^ This is particularly important considering the current uses of generative language models in Akkadian material, with a particular note that such methods have been used to explore metaphors that use words relating to parts of the body.^21^

## Resource availability

### Lead contact

Requests for further information and resources should be directed to the lead contact, Juha M. Lahnakoski (j.lahnakoski@fz-juelich.de ORCID: 0000-0002-5223-7822).

### Materials availability

This study did not generate new materials.

### Data and code availability

• All the data in the current paper are publicly available at https://doi.org/10.5281/zenodo.11242728 with references to external data sources.
• All the code used in the current paper are publicly available at https://doi.org/10.5281/zenodo.11242728.
• Any requests for additional information or resources should be addressed to the lead contact (j.lahnakoski@fz-juelich.de ORCID: 0000-0002-5223-7822).

## Acknowledgments

This research was conducted as part of the project Embodied Emotions: Ancient Mesopotamia and Today, funded by the 10.13039/501100003125Finnish Cultural Foundation (project no. 00220992). We thank Heidi Jauhiainen for providing example files to test the scripts used to produce the word embeddings. The PMI-embeddings script was developed with the assistance of the Center of Excellence Ancient Near Eastern Empires, funded by the 10.13039/501100002341Research Council of Finland (decision number 352747). The emotion vocabulary is based on the work of the project “Akkadian and Hittite Emotions in Context” (AHEC, funded by the 10.13039/501100001659Deutsche Forschungsgemeinschaft (DFG, German Research Foundation, project no. 495257771). This research could not have been carried out without the richly annotated dataset on the Open Richly Annotated Cuneiform Corpus (Oracc). We would like to thank the Steering Committee of Oracc (Jamie Novotny, Eleanor Robson, Steve Tinney, and Niek Veldhuis), the PIs of the many Oracc sub-projects, and the scores of researchers who were involved in the lemmatization and digitization process.

## Author contributions

Conceptualization, all authors; methodology, software, validation, formal analysis, investigation, resources, data curation, and writing—original draft, J.M.L. and E.B.; writing—review & editing, all authors; visualization, J.M.L.; supervision and funding acquisition, L.N., M.S., and S.S.

## Declaration of interests

The authors declare no competing interests.

## STAR★Methods

### Key resources table

**Table:** 

REAGENT or RESOURCE	SOURCE	IDENTIFIER
**Deposited data**		

	https://zenodo.org	https://doi.org/10.5281/zenodo.11242728

**Software and algorithms**		

MATLAB R2023b, MathWorks Inc., MA, USA Python (https://www.python.org/)	https://zenodo.org	https://doi.org/10.5281/zenodo.11242728

### Method details

#### Neo-Assyrian corpus

The largest project that stores open, lemmatised Akkadian texts is the Open Richly Annotated Cuneiform Corpus (Oracc; http://www.oracc.org). Thousands of texts written in cuneiform have been manually lemmatised and made openly available, but the time period that is currently best represented in Oracc is the Neo-Assyrian period (c. 934–612 BCE).

We retrieved Neo-Assyrian texts from Oracc in September 2022 (see https://doi.org/10.5281/zenodo.11242728; To reduce the size of the supplementary material, the unprocessed texts are not included in the repository. The repository contains the files of the processed data after each of the processing steps that can be used to reproduce the current results and, for reference, the scripts used. A small set of example texts and a description of the procedure required to download and process a set of texts are included in the Supplementary file set for reference.). We took a maximalist approach to have as large a dataset as possible. We selected all texts that included the following metadata tags (as defined by Oracc).

(1) ‘Akkadian’; ‘akkadian’; ‘Akkadian with Sumerian incipits’; ‘Akkadian, Aramaic?’; ‘Akkadian, with Aramaic epigraph’; ‘Akkadian?’; ‘Assyrian’; ‘Akkadian, Aramaic’
(2) ‘Neo-Assyrian’; ‘9th/8th century’; ‘8th/7th century’; ‘9th century’; ‘7th century’; ‘8th century’

The resulting corpus consists of 7,696 texts, and 1,014,890 tokens (words). Each word is represented in the format ‘lemma[guideword]EPOS’. The lemma is the form of the word found in the Concise Dictionary of Akkadian (CDA), the guideword is the first translation given in the dictionary, and EPOS is the effective part of speech that gives the basic grammatical form of the word in its sentence. This information has been manually annotated by many different individuals across the Oracc projects. This format standardises the many different spellings of Akkadian words into a single comparative format that allows for easier semantic analysis. Those words with no back-end data (due to breaks in the text or an uncertainty of the researcher as to the best information to allocate to the word) are represented as ‘_’.

#### Word selection

To study embodied emotions in the selected Neo-Assyrian dataset, we next collated a list of words used to express emotions and parts of the body. Emotion words in Akkadian are currently under intense lexicographical study, particularly in the ongoing project “Akkadian and Hittite Emotions in Context*”* (AHEC; https://www.ao.altertumswissenschaften.uni-mainz.de/akkadian-and-hittite-emotional-concepts-in-context/). The project combed through and identified words that could be used to express emotions in the most important Akkadian dictionaries: the Chicago Assyrian Dictionary (CAD), the Akkadisches Handwörterbuch (AHw), the Concise Dictionary of Akkadian (CDA), and the (Electronic) Supplement to the Akkadian Dictionaries ((E)SAD). More specifically, AHEC collected Akkadian terms (verbs, nouns, adjectives, adverbs) based on dictionary searches in the existing Akkadian dictionaries (AHw, CAD, CDA, DAW) which are in English and German (Akkadian – English, Akkadian – German, German – Akkadian), combined with words collected in previous studies of Akkadian emotion terms.^16^^,^^33^ Dictionary searches were undertaken by looking for Akkadian correspondences to the English/German target words (e.g., anger, angry) or words that are semantically related (e.g., rage, wrath). This process identified the words in these dictionaries that could be used to express a selected group of focal (cross-linguistically attested) emotions. The researchers grouped the lexemes into 13 emotional categories (Table 1) based on their observations. Some of the emotion categories also included sub-categories, which were handled as separate emotion categories in the current study, leading to a total of 18 categories. The researchers at AHEC graciously shared the document of emotion terms and categories with us in September 2022, and we compiled it into a spreadsheet for further processing (see https://doi.org/10.5281/zenodo.11242728). We took a maximalist approach to this word list, and included both words that have a direct emotional meaning (such as ‘ezēzu[be(come)-angry]V’), as well as those that were used in more complex idiomatic expressions to convey emotions (such as ‘petû[open]V’).

In the work of AHEC, emotion words and expressions focused on a set of 13 emotions that are often studied in comparative emotion research and considered to be cross-culturally relevant and representative of the emotion domain (see e.g.,^34^) and that were studied in previous body map studies.^1^^,^^4^ These are: *sadness, anger, joy/happiness, fear/anxiety, hate, love, disgust, compassion/pity, envy/jealousy, pride, surprise, shame/embarrassment,* as well as *sexual arousal/desire* the latter of which was selected as an additional embodied feeling state linked to positive emotion. Due to interrelations between words falling into these emotion categories, the category numbering also reflects this hierarchy (e.g., 1a–c are parts of a broader category). However, in the current work, the subcategories were considered independently, increasing the total number of categories to 18. Each word in the dataset is assigned to an emotion category that the AHEC team deemed was the most associated with the word at the time they shared the document. This is referred to here as the ‘Primary Emotion Category’. Detailed meanings of some words were context specific. In other words, Akkadian emotion terms often have several meanings or nuances that may span varieties of a broad emotion category. For example, a term belonging to the *Anger* family/category may cover the nuances of *Anger, Irritation*, or *Fury*. Additionally, a term may colexify different emotions and span more than one category. For example, a term may stand for both *Grief* or *Worry/Anxiety*, depending on the context). A concrete example is the word *‘*ulṣu[pleasure]N*’*, which could be used to express *Joy*, but also the nuance of *Pleasure* and specifically *Sexual Pleasure*. To reflect these multiple shades of meaning, we also included a Boolean section where each column represents each of the emotional categories or sub-categories identified by AHEC, and a cell has ‘Yes’ if the Akkadian word can be used to represent that emotion. Therefore, the word *‘*ulṣu[pleasure]N*’* would have a ‘Yes’ under both columns *‘*3. c) Pleasure (as part of Joy)*’* and *‘*13. Sexual arousal*’*. However, in the current results, the results are visualized only based on the primary emotional category. This means the words are assigned to the category deemed the most frequently attested according to the document with detailed philological notes received by AHEC. It should be noted that, due to the current state of researching emotions in Akkadian texts, several of these categories are not very well understood and are under-studied (such as category ‘11. Surprise’).

There is currently no complete list of Akkadian words that express parts of the body, although numerous Akkadian anatomical terms are discussed in the Semitic Etymological Dictionary (^35^, pp. 355–359). We therefore had to create our own list. We followed the methodology established by AHEC and retrieved as many words relating to the body in the CDA dictionary as possible. Specifically, we went through the whole CDA dictionary and manually selected all words that translated to names of body parts (e.g., lungs) or words that are used to refer to a body part in Akkadian (e.g., bellows) but excluded words for bodily functions or bodily fluids. This word selection procedure resulted in a total of 442 words of interest, 331 indicating emotions and 111 relating to the body (for a full word lists as well as reasons for the word exclusions see the Master Word List Excel file in https://doi.org/10.5281/zenodo.11242728).

#### Word embeddings

Word embeddings are used commonly in modern corpus linguistics, but explorations into word embedding approaches with Akkadian corpora have been limited thus far. Experiments with algorithms like Word2Vec and FastText have provided results which are similar to a word co-occurrence approach alone, likely partially due to the unique challenges of Akkadian corpora in comparison to the larger training corpora from modern languages used for these algorithms.^9^^,^^36^ However, the PMI-embeddings toolkit addresses the unique issues of Akkadian corpora for this purpose (https://github.com/asahala/pmi-embeddings). For example, the Akkadian corpora have repetitive texts, with similar passages repeated across multiple texts, which skew statistical methods and yield biased results for the language.^19^ Akkadian is also a low-resource language, with limited training data, so many of the more complicated word embedding algorithms or vector space analysis methods – which are designed on very large datasets like English Wikipedia – are not applicable.^37^

The PMI-embeddings script uses co-occurrence data as the basis of word embeddings. In the first stage, it generates pointwise mutual information (PMI) co-occurrence data for every word in the textual corpus. The first stage involves generating a co-occurrence matrix, which required special attention to two (out of the nine) parameters of the script: ‘window_size’ and ‘min_count’. The ‘min_count’ parameter means any words that occur less than that parameter will not be counted in the first co-occurrence table. We set this parameter to 1 to ensure all words in the corpus were taken into account at this stage. The second parameter, ‘window_size’, refers to how we define a co-occurrence. In modern languages like English, co-occurrence studies investigate what words in a sentence occur alongside a target word. This is achieved by identifying punctuation within the text, such as full stops. However, Akkadian does not use punctuation marks. Whilst a general rule is that verbs appear at the end of a sentence, this is not the case in every text (and not every text has a verb preserved). Scholars investigating co-occurrences in Akkadian datasets therefore prefer to use window sizes to define the idea of how ‘close’ a word is to the target word.^7^^,^^9^^,^^20^^,^^36^^,^^38^ In effect, this method ignores syntax, which runs the risk of identifying false collocates that are actually words that span sentences or line breaks in the texts. This can be minimised by choosing a relatively small window size. For our study, we used a window size of 3 words on either side of the target word to minimise this effect.

A matrix of PMI scores for target words is then generated based on this co-occurrence matrix. The higher the PMI score, the more likely the two words are to co-occur together in the text. A score of 0 indicates an independent relationship between the words, and words that seem to repel each other received negative scores. For this stage of the process there were four parameters. In order to focus only on the statistically relevant co-occurrences, we set one of these parameters called ‘threshold’ to 5 (the default value of the script). Together with the parameter ‘shift_type’ (set to 0), this parameter means the script disregards the scores of words that have a score of minus 5 or lower in the co-occurrence matrix. In practice, this filtered out rarely occurring words such as ‘tēgimtu[anger]N’, which only occurs once in the dataset, as they generate very low scores.

Once the PMI matrix is generated, the matrix is factorised through Truncated Singular Value Decomposition,^8^^,^^39^^,^^40^ which reduces the matrix into much fewer dimensions. We chose 60 dimensions, which is a default choice in the PMI-embedding script and has been found to be appropriate for the Oracc corpus (https://docs.google.com/document/d/1TjVWqrhalCDjkOQf-JLk1jmC6N83MWGUIEVjbJpm9Es). This process aims to retain the information of the co-occurrences of word pairs in the cosine distances of the vector pairs (^8^; https://github.com/asahala/pmi-embeddings). The final stage of the script is saving the results as a.vec file, and during this process any empty vectors (such as those for words that received a PMI score of less than minus 5) are discarded. 56 emotion words and 20 body words were discarded through this process, leaving a total of 275 emotion words from 18 primary emotional categories (Table 2), and 91 body words for the next stage.

This method enables a computationally efficient comparison of word co-occurrences and, in the current study, allows for an examination of whether Akkadian words used to express emotions occur in similar contexts to words related to the body.

#### Visualisation of body maps

To create the three-dimensional body maps, we used the models from the BodyParts3D project (https://lifesciencedb.jp/bp3d/;^41^). The steps to create the body maps are summarised in Figure 1; this process was repeated for all 275 emotion words. First, to match the Assyrian words with 3D maps of body parts, we manually searched for the body part lists of both the “IS-A” and “PART-OF” hierarchies of the BodyParts3D library based on synonyms (e.g., backbone – spine) and constituent parts (e.g., vertebra – spine) of the English translations of the anatomical terms identified in the Neo-Assyrian corpus.

As the 3D models of the BodyParts3D project used in the current study (see below) did not include female sex organs, we excluded those words from the current analyses, but included words associated with male sex organs. Additionally, to better distinguish between distinct body parts in the resulting body maps, we excluded body words referring to systems and organs spanning the whole body (e.g., *‘ušultu[vein]N’*) or were broader than more specific body parts (*limbs* vs. *arm* and *leg*) and words that did not unambiguously correspond to a specific body part (e.g., *‘libbu[interior]N’*). However, the skin was used as a mask for visualisation to distinguish the outline of the body. After exclusion, the list of body words included 63 items (including the skin).

We then loaded the matching 3D meshes into MATLAB (version R2023b, The MathWorks, Inc., Natick, MA, USA) using the readObj-function written by Bernard Abayowa (https://www.mathworks.com/matlabcentral/fileexchange/18957-readobj). To reduce the computational requirements, we converted the 3D meshes of the body part models to low-resolution voxel representation in 141 × 101 × 348 (width x depth x height) logical 3D voxel arrays using the Mesh Voxelization tool (v.1.20.0.0; https://se.mathworks.com/matlabcentral/fileexchange/27390-mesh-voxelisation/). For visualisation, the arrays were smoothed by a Gaussian kernel (MATLAB *smooth* function, isotropic 2-voxel standard deviation, kernel array size 9 × 9 × 9 voxels). The 3D images were averaged over depth dimension (chest–back) to produce 2-dimensional images, which were enlarged for visualisation to 631 x 1562 resolution.

While visualising the combined body maps, we assigned the cosine similarity of the point-wise mutual information embedding of the body part and the target emotion (pairwise similarity between the vectors of the body word and the emotion word) as the value of the body part by multiplying the logical body part model with the similarity value. For each voxel where multiple body parts overlapped (e.g., synonyms for the same body part), we selected the (positive or negative) extreme value of the overlapping body parts as the similarity value for the voxel. For the 2D visualizations, we similarly selected the extreme values over the z axis (front to back) as the pixel similarity value in the images. These similarity values of emotion-vs. body-word pairs were mapped to a color scale to produce maps where highly similar word pairs in the 60-dimensional embedding space (see the previous discussion) were associated with hot (red) colors. Inverse associations of the word embeddings (negative correlation, suggesting an occurrence of one word consistently predicts the absence of another one in a similar context) were associated with cold (blue) colors. Black colors indicate a lack of statistical association (zero correlation). In the figures, dot and breve diacritic marks under the letters ṭ, ṣ and ḫ have been replaced where necessary due to character encoding issues (‘ṭ’ with ‘t.’, ‘ṣ’ with ‘s.’ and ‘ḫ’ with ‘h_’).

To simplify the visualisation of the emotional space on the body, in addition to individual emotions, we calculated the mean of the word vectors of all emotions belonging to the larger emotion categories and evaluated the similarity of this mean vector and the body parts. This produced a single body map for each of the 18 emotion categories. For the sake of comparison, we also created body maps for individual emotions (for the number of emotions in each category, see Table 1) and then averaged those for each emotion category. The resulting body maps were similar with both approaches. Therefore, we focus only the maps for the mean emotion vectors of each emotion category in the results section.

### Quantification and statistical analysis

The current results show relative semantic distances based on cosine similarities. There are no quantification or statistical analyses to include in this study.

## Supplemental information

Data S1. Procedure to download and process a set of texts including an example set of texts
