# Embodied emotions in ancient Neo-Assyrian texts revealed by bodily mapping of emotional semantics

**DOI:** 10.1016/j.isci.2026.115347

**Published:** 2026-03-14

**Authors:** Juha M. Lahnakoski, Ellie Bennett, Lauri Nummenmaa, Ulrike Steinert, Mikko Sams, Saana Svärd

## Main text

(iScience *27*, 111365; December 20, 2024)

During proofing, an error was introduced in Table 2 of the published article. Specifically, the term “eṣemṣeru[ankle]N” was used in row 1, column 1, instead of the correct term, “kiṣallu[ankle]N.” This Akkadian word is found elsewhere in the table with its correct translation (eṣemṣeru[backbone]N). The authors and editors apologize for this error. The table has been updated in the original article.

